# Cats at the Vet: The Effect of Alpha-s1 Casozepin

**DOI:** 10.3390/ani10112047

**Published:** 2020-11-05

**Authors:** Adjet Makawey, Christine Iben, Rupert Palme

**Affiliations:** 1 Department of Farm Animals and Veterinary Public Health, Institute of Animal Nutrition and Functional Plant Compounds, University of Veterinary Medicine, Veterinärplatz 1, 2210 Vienna, Austria; Christine.Iben@vetmeduni.ac.at; 2 Department of Biomedical Sciences, Unit of Physiology, Pathophysiology and Experimental Endocrinology, University of Veterinary Medicine, Veterinärplatz 1, 2210 Vienna, Austria; Rupert.Palme@vetmeduni.ac.at

**Keywords:** stress, feces, cortisol metabolites, non-invasive, Zylkène, behaviour

## Abstract

**Simple Summary:**

Cat owners try to avoid necessary health checks at the vet, because they are related to stress for both the cat and them. In this study, we examined the influence of the dietary supplement, α-s1 casozepin, on fecal cortisol metabolites and the autonomic nervous system in cats. We found evidence for a positive effect on the autonomic nervous system. Our findings, although preliminary, suggest that α-s1 casozepin may improve the coping of cats with stressful events.

**Abstract:**

The aim of this study was to evaluate the impact of α-s1 casozepin on cat stress responses at a veterinary practice. Cats feel confident in their familiar surroundings and daily routine. A visit, and transport, to the veterinarian is a stressful experience for cats and their owners. Stress can mask clinical signs and has physiological impacts. Alpha-s1 casozepin (Zylkène; Vétoquinol) could potentially minimize stress in cats with its calming and anxiolytic characteristics. A randomized, partial double-blind and placebo-controlled study was carried out with 60 adult cats. The trial was designed for three groups: low dose (15 mg/kg q24 h α-s1 casozepin for six days), high dose (75 mg/kg q24 h α-s1 casozepin for three days), and a placebo (one fructose capsule per day for three days). For the study, cats had a checkup at their trusted veterinarian without the dietary supplement, followed by a second one four weeks later. Alpha-s1 casozepin or a placebo was administered three to six days before the checkup. Fecal cortisol metabolites (FCMs) were measured to non-invasively evaluate the impact of α-s1 casozepin on adrenocortical activity. The cat owners and veterinarians also assessed the physiological reactions (respiratory rate, sweaty paws, pupils, panting, and vocalization) of the cats at home, in the waiting area, and in the examination room. The only significant effect (kappa coefficient κ = 0.007 and κ = 0.003) found in this study was the absence of sweaty paws in cats who were treated with the high dose of α-s1 casozepin over three days, observed in the waiting area and examination room of the veterinarian’s practice, respectively. Alpha-s1 casozepin also showed a small but insignificant reduction in FCM levels. Alpha-s1 casozepin influences the autonomic nervous system, and can inhibit sweaty paws during stressful situations for cats.

## 1. Introduction

Domestic cats are creatures of habit. Changes in their daily routine and environment can potentially cause stress [1,2,3,4]. Cats need regular veterinary checkups throughout their lives to ensure early detection of any health problems, and a high quality of life [5]. Therefore, an inevitable feline experience is a regular examination at the veterinarian, making it impossible for cats to permanently avoid unfamiliar, new, or stressful situations. Most cats experience visits to a veterinary practice as anxiety-inducing and stressful [2,5,6,7,8,9]. From the cat owners’ point of view, taking the cat to the vet is highly stressful for the cat and themselves [5,6,7,8,9]. Cats may react aggressively when being transported to the vet or react defensively to avoid having to go [2,5,6,7,8]. Therefore, owners may forego medical checks and vaccinations in an effort to not distress their pets [7]. The consequence is too often a lack of medical care for the pet cats. Moreover, with cats that do go to a vet for treatment, impaired behaviour may conceal signs of disease, and cause difficulties with their clinical examination [9,10].

Veterinarians here in Austria sometimes administer bovine α-s1 casozepin for its calming and anxiolytic effects [11]. As a nutraceutical it is classified as food, and absolutely safe to use. Therefore, pet owners do not need to have any concerns about addiction potential [12]. Behaviourists appreciate the positive effect on cats with anxiety disorders [13]. Several animal species benefit from its behavioural and physiological effects. Anxious dogs’ behaviour and serum cortisol levels significantly decreased by feeding caseinate hydrolysate in the diet [14]. Calming effects of α-s1 casozepin in stressful situations, such as handling, training, and loading of horses for transport were reported [15,16]. Beata et al. showed that α-s1 casozepin had a positive effect on cats with anxiety disorders [17]. Milk proteins consist of numerous bioactive peptides, which may be released after enzymatic hydrolysis. Alpha-s1 casozepins are peptides and tryptic hydrolysates of bovine α-s1 casein (f91–100). They display an affinity for the GABAA receptor (γ-amino-butyric acid), and cause anxiolytic, anticonvulsant, anti-thrombotic, opioid, and anti-stress effects [13,18,19]. Similar effects were shown by an experimental study in rats, where diazepam and α-s1 casozepin were equally effective [20].

Although there is no single parameter to define and measure stress, it does in fact play a critical role in animal welfare [21], meaning different parameters should be taken into account for its evaluation. The adrenal glands are key in the hormonal response of stress. In pressure situations, they react with an increase of glucocorticoid and/or catecholamine secretion, which is among the first defence reactions to stress [21]. The autonomic nervous system responses include increased heart rate, blood pressure, and sweaty paws [22,23]. The current state of research provides no information about the effect of α-s1 casozepin on the adrenal cortex of cats. Therefore, the aims of this study were first, to evaluate a possible effect of α-s1 casozepin on fecal cortisol metabolites (FCMs), a non-invasive measure of adrenocortical activity [22,23]. Second, to evaluate a possible impact of α-s1 casozepin on the autonomic nervous system by a simple stress reaction questionnaire. Depending on previous experience with the substance, veterinarians administer different doses of α-s1 casozepin [9,24]. Thus, finally, we also tried to optimize α-s1 casozepin dosages for cats, hypothesizing that a larger, short-term dose would achieve a stronger reduction in FCM concentrations.

## 2. Materials and Methods

### 2.1. Animals

The study required the recruitment of veterinarians and cat owners, or rather their cats. The majority (90%) of the veterinarians were recruited and instructed in face-to-face meetings, providing a thorough explanation of the study’s procedure. A small proportion (10%) were reached using an announcement in an Austrian journal of veterinary medicine, and through other veterinarians. Participating veterinarians searched for cat owners, who were willing to support the study without payment, and were able to manage a specific sample collection. This means that the feces of the participating cat must be identifiable and collectable immediately after defecation. All veterinarians were interested in expanding their knowledge about α-s1 casozepin with this study. They were provided with the required sample material and obtained a personal description of the study design. In the study, they were responsible for instructing their owners, and carried out the respective clinical examination.

The recruitment of the cats took place either in direct communication with their owners, or cooperating veterinarians. Various methods were used to contact owners: privately via family, friends, and their contacts (email, WhatsApp, telephone, meetings), public tenders in Facebook groups for cat owners, and on the websites of veterinary practices.

This study examined animals that were housed indoors, to ensure correct and punctual feces sampling. All cats used their litter box with their familiar litter brand to ensure normal defecation behaviour and avoid additional stressors. Exclusion criteria during the trial were (a) planned travelling, (b) planned household relocation, or (c) other predictable disturbances, e.g., renovation work, parties, and enlargement of the family (children or pets). Furthermore, cats which were treated with glucocorticoids, α-s1 casozepin, or which suffered from adrenal diseases were also excluded. All participating cats were identified as not having a manifest allergy or intolerance, but cow’s milk allergy or intolerance were not excluded by a blood test. Cooperating veterinarians ensured that during the experiment, all cats were healthy and had no identifiable behavioural disorders, e.g., reduced feed intake, overgrooming, urine marking, and some forms of aggression. Their last veterinarian visit was more than four weeks ago. Anxiety disorders were not excluded by a veterinarian of behavioural medicine. This experiment was discussed with and approved by the institutional ethics and animal welfare committee, in accordance with GSP guidelines and national legislation (ETK-23/03/2016).

### 2.2. Experiment

**Figure 1:**
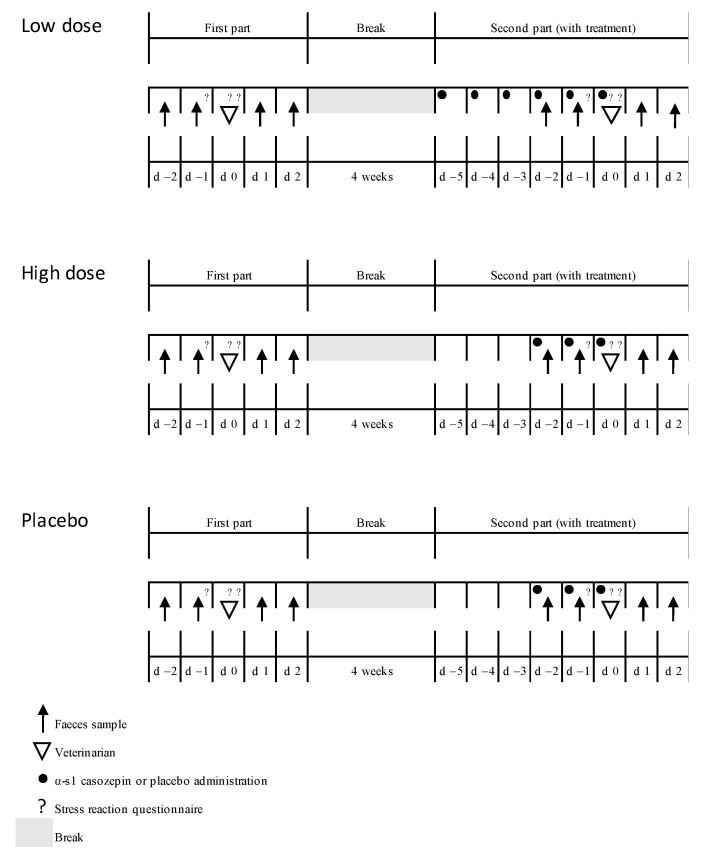
Timeline of the experiment.

The experiment’s procedure was outlined (see Figure 1 for an overview) in detail using handouts for the veterinarians and cat owners. The sample material consisted of a freezer bag and a folder. The lockable freezer bags contained eight plastic containers and eight wooden spatulas. The folder included the owner’s consent form, a detailed description of the feces collection and storage, a form for personal details about the cat, two sheets of the stress reaction questionnaire, two checklists for the clinical investigations, and the capsules.

A randomized, partial double-blind and placebo-controlled study was designed for three groups (20 cats each): low dose (LD), high dose (HD), and placebo (P). The study was divided in two equal parts with a break of four weeks in between. The second part varied from the first part only by the α-s1 casozepin (or placebo) administration (see Figure 1). Cats in the LD group received one capsule with 15 mg/kg α-s1 casozepin dosage (75 mg α-s1 casozepin q24 h up to 5 kg body weight) over a period of six days. This dosage was recommended by the manufacturer, and was therefore not placebo-controlled. Cats in the HD group received one capsule with 45 mg/kg α-s1 casozepin dosage (225 mg α-s1 casozepin q24 h up to 5 kg body weight) over a period of three days. Cats in the P group received the placebo over a period of three days.

The α-s1 casozepin 75 mg white/blue capsules consisted of 77.3% powder (trypsin hydrolysed bovine casein, maltodextrin, magnesium stearate) and 22.7% capsule (bovine hide gelatin). The α-s1 casozepin 225 mg blue capsules consisted of 76.9% powder (trypsin hydrolysed bovine casein, maltodextrin, magnesium stearate) and 23.1% capsule (bovine hide gelatin). The placebo consisted of sucrose powder and was supplied by the pharmacy at the University of Veterinary Medicine, Vienna. The α-s1 casozepin 225 mg capsules and the placebo were administered in exactly the same capsule form and colour. The α-s1 casozepin 75 mg capsules were not administered placebo-controlled, and were distinguishable by the blue colour and length of the administration.

In detail (for an overview see Figure 1), the trial began two days before the first visit to the vet. On these days (d-2 and d-1) a plum-sized amount of feces was collected in plastic containers and frozen at −18 °C within 30 min after defecation. These samples were used for baseline measurement. The day before the consultation (d-1), the owners completed the cats’ stress reaction questionnaire during a phase where their cat was calm. The stress reaction questionnaire (Table 1) was especially designed for this study by the authors and Sabine Schroll, an Austrian behavioural scientist and veterinarian for cat medicine. Simple questions were selected for cat owners to observe changes and responses. It should be mentioned that this questionnaire was not validated before. The stress reaction questionnaire was filled in, (1) at home, (2) in the waiting area, and (3) in the examination room. It was completed in the first and the second part of the trial in the same way, and always contained the same questions (see Figure 1).

The checkup was performed at the cats’ veterinarian on day 0. Cats were transported in their animal carriers by their owners. Depending on the distance, the owners used different means of transport, arriving at the veterinarian’s practice by car, bus, underground, tram, or on foot. The owners completed their cats’ stress reaction questionnaire in the waiting room. The stress reaction questionnaire was completed again in the examination room, starting with the item “cat exits the transport cage on its own (within 1 min)”.

Afterwards, the clinical examination was performed as a general examination to assess the health condition of the cat. The examination involved the consideration of (1) physical body condition, (2) nutritional condition, (3) skin and hair coat, (4) hydration status, (5) eyes, (6) nose and nares, (7) oral cavity, (8) peripheral lymph nodes, (9) pulse, (10) cardiac auscultation and heart rate, (11) respiratory auscultation and respiratory rate, and (12) abdominal palpation. The cat’s body temperature was not measured. If any problems or diseases were detected, the cat was excluded from the study. Fecal samples were collected and immediately frozen on days 1 and 2. The second part of the study commenced following a break of four weeks. Four weeks were expected to be an adequate length to recover from the first visit at the veterinarian’s practice [25]. The cats were not exposed to any unusual disturbances during this break period (e.g., being kennelled, construction work in their home environment, new pet in the household). The second part began with the administration of α-s1 casozepin. Group LD started on day-5, and groups HD and P on day-2. The capsules were administered directly into the mouth or blended into a small amount of wet food before the cats were fed their breakfast. The cats were dosed once per day in the morning. The trial was continued using the same procedure as applied in the first part. Feces were collected and frozen on days-2 and -1. The same stress reaction questionnaire was also logged on day-1 at home, and on day 0 in the waiting and examination rooms. On day 0 the cats in each group had their second examination at their veterinarian, arriving via the same method of transportation used four weeks prior. Feces were collected and frozen on days 1 and 2. If cats did not defecate daily, the next two samples were collected starting from day 1.

### 2.3. Determination of Fecal Cortisol Metabolites

A total of 0.5 g wet feces from each well-homogenised sample was extracted with 5 mL methanol (80%) [26]. An aliquot of the supernatant was measured with an 11-oxoaetiocholanolone enzyme immunoassay (EIA). This EIA (measuring 11,17-dioxoandrostanes, a group of cortisol metabolites) was first described by Palme and Möstl, and successfully validated for use in cats [26,27,28].

### 2.4. Statistical Analysis

The data were presented on spreadsheets, and with graphics using descriptive statistics. FCM values were log transformed to comply to a normal distribution. Analysis of variance (ANOVA) was applied to compare FCM levels from the first and second part of the experiment. Significant differences between the mean values of each group (LD, HD, and P) were tested via kappa coefficient. Post-hoc tests were applied in case of multiple comparisons. Statistical analyses were performed with IBM SPSS Statistics 25, version 16.

## 3. Results

### 3.1. Data about Cats

A total of 60 neutered adult cats (30 of each sex) were selected from Vienna and its surrounding vicinity. The cats were between one and eighteen years old (mean: 7). No cat opted out during the study, although 11 fecal samples could not be analysed. One sample was not completed, two samples had already dried up and eight samples had abnormally low FCM values (below the detection limit of 4.4 ng/g). Hence the stress reaction questionnaire included the complete sample population of the cats (*n* = 60), and the analysis of the fecal samples with the EIA excluded 11 of the population (*n* = 49). The cats’ characteristics are shown in Table 2.

### 3.2. Stress Reaction Questionnaire

The owner of each cat filled out the stress reaction questionnaire in different surroundings. Cats were observed without treatment (first part) and four weeks later under administration of α-s1 casozepin (second part). Cats were observed at home to check them within their familiar environment. Regardless of the group, the entire population showed an “unstressed” stress reaction questionnaire in both parts. In the waiting room two cats from HD and one cat from LD saw their respiratory rate improve under treatment. Values from P remained constant. The respiratory rate was not significantly influenced by any treatment group.

In the waiting room (Table 3), five cats no longer had sweaty paws under HD treatment. Cats which did not show sweaty paws in the first part of the study continued to not have them in the second part, also under HD treatment. Cohen’s kappa coefficient revealed a significant result in the HD group (i.e., there was no correlation between the first and second parts, κ = 0.007). Improvement under LD treatment was shown in seven cats who no longer had sweaty paws. Three cats had sweaty paws in the second part. The pupils of the cats, panting, and vocalization were significantly influenced neither by treatment nor placebo.

In the examination room three cats from HD and LD improved their respiratory rate in the second part. However, the respiratory rate deteriorated for one cat in HD and P. Treatment had no significant effect on the respiratory rate.

In the examination room (Table 4), four cats in HD no longer had sweaty paws. Five cats no longer had sweaty paws under LD treatment, and one cat exhibited sweaty paws. Three cats no longer had sweaty paws under P treatment. However, four cats, also under P treatment had sweaty paws in the second part. Cohen’s kappa coefficient revealed a significant result in the HD group (κ = 0.003). There was no correlation between sweaty paws in the first and second parts in HD group. Cohen’s kappa coefficient furthermore recorded no correlation between the first and the second parts in LD (κ = 0.095), but a correlation in P (κ = 0.178). The treatment had no effect on panting, the pupils of the cats, vocalization, nor did treatment affect the cats exiting their transport carrier.

### 3.3. Fecal Cortisol Metabolites

**Figure 2:**
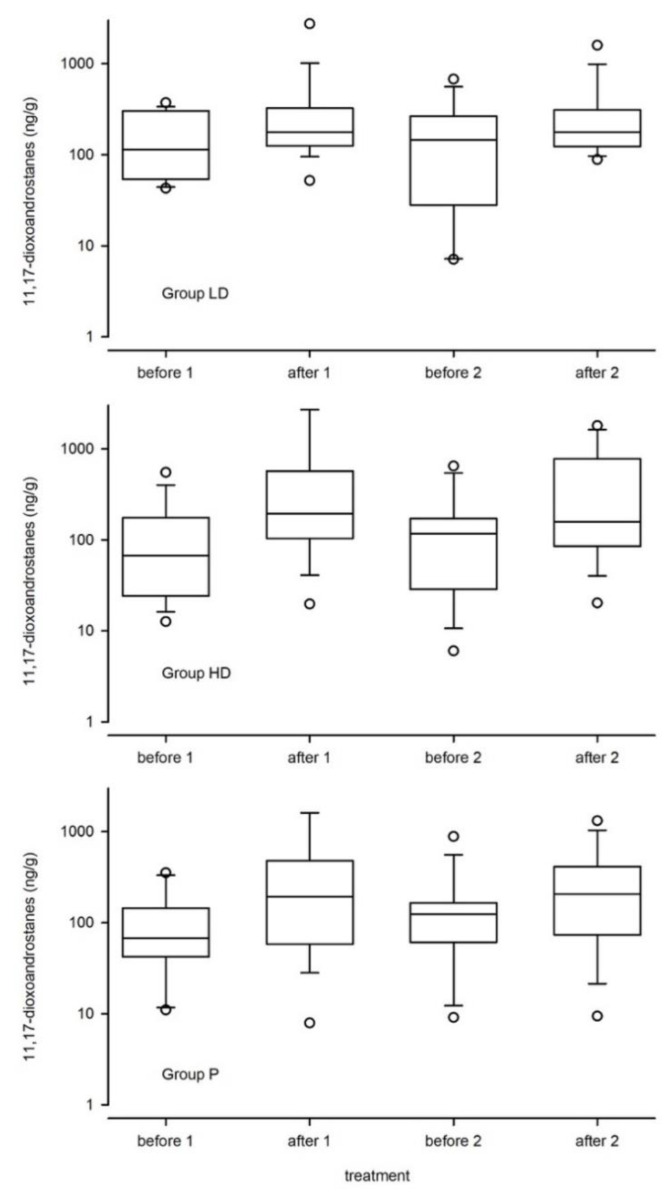
Effects of α-s1 casozepin in different doses (LD = low dose; HD = high dose; P = placebo) and times on fecal cortisol metabolites (11,17-dioxoandrostanes; note the logarithmic scale of the y-axes).

The boxplots (Figure 2) of FCM levels compare the three groups before and after the visit at the veterinarian (stressor). Values from the first part, without treatment were described as before 1, and after 1. Values from the second part, with LD, HD, and P treatment, were described as before 2, and after 2. FCM levels increased significantly (*p* = 0.001) after stressor exposure in all groups. The treatment resulted in a small, but insignificant reduction of FCM levels. This effect was not affected by the dosage. Animals in all groups showed marked individual differences in FCM concentrations. Levels in group LD had the least variation.

## 4. Discussion

There are various approaches attempting to reduce cats’ stress when visiting the veterinarian [29,30,31]. The aim of our practice-oriented study was to suggest a feasible solution for these cats and their owners. Our results showed that bovine α-s1 casozepin had a small impact on the autonomic nervous system of the cats in the experiment, and a small, but insignificant, effect on their adrenocortical activity.

Beata et al. [17] used α-s1 casozepin for the treatment of cats with anxiety disorders over a period of eight weeks. Cats here were evaluated three times using the cat emotional scale. Treated cats showed a significant improvement in their relationship with familiar and unfamiliar people, albeit with unchanged fear reactions. A combination of α-s1 casein and L-tryptophan was found in another study to increase the activity of anxious cats. Important to note here was that they were laboratory-housed for the study [24]. The same ingredients were used for a special diet to reduce the signs of feline idiopathic cystitis. The “cat emotional and quality of life score” improved significantly after the diet was administered for eight weeks [32]. Other studies [4,6,8,33], focusing on stress and behaviour, used the cat-stress-score (CSS) designed by Kessler and Turner [33].

In contrast, cats in our study were in a generally healthy condition. In our study, the effect of α-s1 casozepin was examined by a stress reaction questionnaire, and by evaluating adrenocortical and autonomic nervous system activity to examine visible signs of stress. The fact that the participating cats stayed in their owner’s households during the entire trial and were examined by their trusted veterinarians kept the study as close to reality as possible. This was a result of the high compliance by the cat owners and veterinarians. The stress reaction questionnaire was selected to allow a simple procedure for cat owners [30]. The results of the questionnaire “at home” suggested that the treatment had no effects on cats when they were not exposed to stress. Further results indicated that a significant number of cats did not display sweaty paws under α-s1 casozepin treatment. Consequently, α-s1 casozepin appears to influence the autonomic nervous system [22,23,34].

In healthy humans, alpha-s1 casozepin was shown to decrease cortisol concentrations significantly [19]. Palme et al. [35] investigated the effect of a vaccination procedure on FCMs in laboratory-reared cats and dogs, finding a significant increase in FCM concentrations after a ten-minute transport by car and vaccination in a veterinary practice. Major individual differences were recorded in concentrations of FCMs, and the time to reach peak levels. They also found that the lag time of FCM excretion may vary depending on cats’ activity (and defecation rate) [35].

However, our study could not find a significant influence of α-s1 casozepin on fecal cortisol metabolites in cats. Similarly to Palme et al. [35], we found an increase in FCM levels after the visit to the vet, whether under α-s1 casozepin or placebo treatment. We also recognized a large variation among individuals, both in baseline as well as maximum FCM values.

Schroll and Dehasse recommend 125 mg/cat q24 h α-s1 casozepin for cats, which are difficult to handle [11]. Schroll [36] recommends different indications and doses for dogs and cats with behaviour disorders, or those frightened by stressful events. In such cases, cats were treated with between 125 to 225 mg/cat q24 h α-s1 casozepin. The period of the administration depended on the individual case and was from three days to four weeks or longer [36]. Celina del Amo used α-s1 casozepin against anxiety disorders in doses of 15 mg/kg for at least 10 to 14 days before the stressful event [37]. These recommendations comply with our study’s results, with cats from the HD and LD group showing significantly lower occurrences of sweaty paws in the examination room. The LD treatment complied with the recommendations of Celina de Amo [37], and the HD treatment was recommended by Schroll [36]. In the waiting room, this effect was only present in the HD group. Important to note is that in the waiting room, cats were exposed to additional stressors (e.g., the transport carrier, other animals, and unfamiliar people) compared to during the clinical examination, suggesting the higher dose to be more effective. However, detailed information about the waiting room and its features (separated waiting areas for cats and dogs, the presence of other people or pets), and waiting time were not evaluated.

## 5. Limitations and Further Research

The results showed a small but insignificant impact of α-s1 casozepin on FCM levels. Expressed individual variations probably masked the effect of α-s1 casozepin. A larger number of cats for each group could better prove the efficacy of α-s1 casozepin. With this being said, it is important to note that it was difficult to find 60 cats of open-minded owners, who were willing to carefully participate in this study’s trial.

With her long-term experience as an animal behaviourist, Schroll (S. Schroll, 2016, personal communication) assumed that α-s1 casozepin has the best effect on moderately stressed cats. Further studies about cats and their stress management should integrate their personality, because doing this could produce different findings regarding stress responses [38,39].

## 6. Conclusions

Alpha-s1 casozepin significantly inhibited the stress-induced reaction of sweaty paws, probably by influencing the autonomic nervous system. In the case of HD treatment, this reduction was present in both the waiting room and examination room. The LD treatment only inhibited the sweaty paws in the examination room. These findings revealed that the difference between a short treatment with a higher dose and a long treatment with a lower dose is minimal. In conclusion, an administration of α-s1 casozepin may help reduce stress reactions in cats, improving their well-being as a result. Due to the limitations of this study, further studies are required to confirm our results.

## Figures and Tables

**Table 1:** Stress reaction questionnaire.

Respiratory rate (>30/min.)	Yes or No
Sweaty paws	Yes or No
Pupils	Small, Normal, Large, or Closed
Panting	Yes or No
Vocalization	
purring	Yes or No
Growling	Yes or No
Hissing	Yes or No
Meowing or squeaking	Yes or No

**Table 2:** Characteristics of participating cats (*n* = 60; 100%).

		Type of Analysis			
		Stress Reaction Questionnaire		EIA	
Characteristics	Categories	*n*	%	*n*	%
Sex	Male	30	50.0	21	42.9
(neutered)	Female	30	50.0	28	57.1
Age	1–6 years	25	41.7	21	42.9
	7–12 years	27	45.0	21	42.9
	>12 years	8	13.3	7	14.3
Breed	Common Domestic Cats	43	71.7	33	67.3
	Purebred Cats	13	21.7	13	26.5
	Crossbred Cats	4	6.7	3	6.1
Housing	single-housed	22	36.7	20	40.8
	multiple-housed	38	63.3	29	59.2
Transport	on foot	21	35.0	17	34.7
	car	31	51.7	25	51.0
	bus	4	6.7	4	8.2
	underground	3	5.0	2	4.1
	tram	1	1.7	1	2.0

**Table 3:** Crosstab “sweaty paws” in the waiting room.

			Second Part		
Group			Yes	No	Total
HD	First Part	yes	7	5	12
		no	0	8	8
LD	First Part	yes	3	7	10
		no	3	7	10
P	First Part	yes	3	3	6
		no	4	10	14
total	First Part	yes	13	15	28
		no	7	25	32
	total		20	40	60

HD: high dose, LD: low dose, and P: placebo treatment.

**Table 4:** Crosstab “sweaty paws” in the examination room.

			Second Part		
Group			Yes	No	Total
HD	First Part	yes	9	4	13
		no	0	7	7
LD	First Part	yes	5	6	11
		no	1	8	9
P	First Part	yes	6	3	9
		no	4	7	11
total	First Part	yes	20	13	33
		no	5	22	27
	total		25	35	60

HD: high dose, LD: low dose, and P: placebo treatment.

## References

[1] Rochlitz I. (2005). A review of the housing requirements of domestic cats (Felis silvestris catus) kept in the home. Appl. Anim. Behav. Sci..

[2] Döring D., Tiefenbach P., Erhard M. (2013). Angst- und stressbedingte Verhaltensprobleme bei der Katze-Eine Übersicht für den praktischen Tierarzt. Kleintierpraxis.

[3] Amat M. (2016). Stress in owned cats: Behavioural changes and welfare implications. J. Feline Med. Surg..

[4] Hampton A., Ford A., Cox R.E., Liu C., Koh R. (2019). Effects of music on behaviour and phyiological stress response of domestic cats in veterinary clinic. J. Feline Med. Surg..

[5] Vogt A.H., Rodan I., Brown M., Brown S., Buffington C.A., Forman M.J., Neilson J., Sparkes A. (2010). AAFP-AAHA: Feline life stage guidelines. J. Feline Med. Surg..

[6] Pereira J.S., Fragoso S., Beck A., Lavigne S., Varejão A.S., da Graça Pereira G. (2015). Improving the feline veterinary consultation: The usefulness of Feliway spray in reducing cats’ stress. J. Feline Med. Surg..

[7] Volk J.O., Felsted K.E., Thomas J.G., Siren C.W. (2011). Executive summary of the Bayer veterinary care usage study. J. Am. Vet. Med. Assoc..

[8] Pratsch L., Mohr N., Palme R., Rost J., Troxler J., Arhant C. (2018). Carrier training cats reduces stress on transport to a veterinary practice. Appl. Anim. Behav. Sci..

[9] Mariti C., Bowen J.E., Campa S., Grebe G., Sighieri C., Gazzano A. (2016). Guardians’ perceptions of cats’ welfare and behavior regarding visiting veterinary clinics. J. Appl. Anim. Welf. Sci..

[10] Quimby J.M., Smith M.L., Lunn K.F. (2011). Evaluation of the effects of hospital visit stress on physiologic parameters in cat. J. Feline Med. Surg..

[11] Schroll S., Dehasse J. (2015). Verhaltensmedizin bei der Katze: Leitsymptome, Diagnostik, Therapie und Prävention.

[12] Beata C., Beaumont-Graff E., Diaz C., Marion M., Massal N., Marlois N., Muller G., Lefranc C. (2007). Effects of alpha-casozepine (Zylkene) versus selegiline hydrochloride (Selgian, Anipryl) on anxiety disorders in dogs. J. Vet. Behav..

[13] Lecouvey M., Frochot C., Miclo L., Orlewski P., Driou A., Linden G., Gaillard J.L., Marraud M., Cung M.T., Vanderesse R. (1997). Two-dimensional ^1^H-NMR and CD structural analysis in a micellar medium of a bovine αs1-casein fragment having benzodiazepine-like properties. Eur. J. Biochem..

[14] Palestrini C., Minero M., Cannas S., Berteselli G., Scaglia E., Barbieri S., Cavallone E., Puricelli F.S., Dall’Ara P. (2010). Efficacy of a diet containing caseinate hydrolysate on signs of stress in dogs. J. Vet. Behav..

[15] McDonnell S.M., Miller J., Vaala W. (2013). Calming benefit of short-term alpha-casozepine supplementation during acclimation to domestic environment and basic ground training of adult semi-feral ponies. J. Equine Vet. Sci..

[16] Ijichi C., Green S., Squibb K., Carroll A., Bannister I. (2019). Zylkéne to load? The effects of alpha-casozepine on compliance and coping in horses during loading. J. Vet. Behav..

[17] Beata C., Beaumont-Graff E., Coll V., Cordel J., Marion M., Massal N., Marlois N., Tauzin J. (2007). Effect of alpha-casozepine (Zylkene) on anxiety in cats. J. Vet. Behav..

[18] Miclo L., Perrin E., Driou A., Papadopoulos V., Boujrad N., Vanderesse R., Boudier J.F., Desor D., Linden G., Gaillard J.L. (2001). Characterization of α-casozepine, a tryptic peptide from bovine αs1-casein with benzodiazepine-like activity. FASEB J..

[19] Messaoudi M., Lefranc-Millot C., Desor D., Demagny B., Bourdon L. (2004). Effects of a tryptic hydrolysate from bovine milk αs1-casein on hemodynamic responses in healthy human volunteers facing successive mental and physical stress situations. Eur. J. Nutr..

[20] Schroeder H., Violle N., Messaoudi M., Lefranc-Millot C., Nejdi A., Demagny B., Desor D. (2003). Effects of ING-911, a tryptic hydrolysate from bovine milk alpha-S1 casein on anxiety of Wistar male rats measured in the conditioned defensive burying (CDB) paradigm and the elevated plus maze test. Behav. Pharmacol..

[21] Möstl E., Palme R. (2002). Hormones as indicators of stress. Domest. Anim. Endocrinol..

[22] Anderson D.J., Adolphs R. (2014). A framework for studying emotions across species. Cell.

[23] Robertshaw D. (1985). Sweat and heat exchange in man and other. J. Hum. Evol..

[24] Landsberg G., Milgram B., Mougeot I., Kelly S., de Rivera C. (2017). Therapeutic effects of an alpha-casozepine and L-tryptophan supplemented diet on fear and anxiety in the cat. J. Feline Med. Surg..

[25] Rudolph D., Kraft W. (2004). Vergleich zweier Bestimmungsmethoden für Schilddrüsenhormone, Kortisol und Insulin bei der Katze. Chemilumineszenz- und Elektrochemilumineszenz-Technik. Tierarztl. Prax. K. H..

[26] Schatz S., Palme R. (2001). Measurement of faecal cortisol metabolites in cats and dogs: A non-invasive method for evaluating adrenocortical function. Vet. Res. Commun..

[27] Palme R., Möstl E. (1997). Measurement of cortisol metabolites in faeces of sheep as a parameter of cortisol concentration in blood. Z. Säugetierkd.

[28] Palme R. (2019). Non-invasive measurement of glucocorticoids: Advances and problems. Physiol. Behav..

[29] Palestrini C. (2009). Situational sensitivities. BSAVA Manual of Canine Feline Behavioural Medicine.

[30] Bradshaw J. (2018). Normal feline behavior …. and why problem behaviours develop. J. Feline Med. Surg..

[31] Sadek T., Hamper B., Horwitz D., Rodan I., Rowe E., Sundahl E. (2018). Feline feeding programs: Addressing behavioural needs to improve feline health and wellbeing. J. Feline Med. Surg..

[32] Meyer H.P., Bečvářová I. (2016). Effects of a urinary food supplemented with milk protein hydrolysate and L-tryptophan on Feline Idiopathic Cystitis-results of a case series in 10 cats. Int. J. Appl. Res. Vet. Med..

[33] Kessler M.R., Turner D.C. (1999). Effects of density and cage size on stress in domestic cats (Felis silvestris catus) housed in animal shelters and boarding catteries. Anim. Welf..

[34] Frank J., Griffin T. (1989). Stress and immunity: A unifying concept. Vet. Immunol. Immunopathol..

[35] Palme R., Schatz S., Möstl E. (2001). Influence of a vaccination on faecal cortisol metabolite concentrations in cats and dogs. Dtsch. Tierarztl. Woch..

[36] Schroll S. Fallbeispiele zu Hund und Katze aus der Verhaltenspraxis. https://www.vet-webinar.com/de/webinare/detail/d/Fallbeispiele_zu_Hund_und_Katze_aus_der_Verhaltenspraxis/202/24/.

[37] Del Amo C. (2011). Einsatz von Arzneimitteln, Futterzusatzstoffen und Futtermitteln bei Angstproblemen. Team. Konkret.

[38] Litchfield C.A., Quinton G., Tindle H., Chiera B., Kikillus K.H., Roetman P. (2017). The ‘Feline Five’: An exploration of personality in pet cats (Felis catus). PLoS ONE.

[39] Fukimoto N., Melo D., Palme R., Zanella A., Mendonça-Furtado O. (2020). Are cats less stressed in shelters? A study of personality and faecal cortisol metabolites. Appl. Anim. Behav. Sci..

